# Perceived Barriers to Exercise in Adults with Traumatic Brain Injury Vary by Age

**DOI:** 10.3390/jfmk3030047

**Published:** 2018-09-14

**Authors:** Shanti M. Pinto, Mark A. Newman, Mark A. Hirsch

**Affiliations:** Department of Physical Medicine and Rehabilitation, Carolinas Rehabilitation, Charlotte, NC 28203, USA

**Keywords:** traumatic brain injury, exercise, physical activity, aging, barriers, International Classification of Functioning, Disability, and Health

## Abstract

Physical activity and exercise are important adjuncts to medical treatment for overall health in individuals with traumatic brain injury (TBI); however, many individuals do not partake in the recommended weekly exercise. The objective of this study was to investigate the barriers to exercise after TBI and determine whether these barriers varied by age. The sample was 172 adults with moderate to severe TBI who completed Barriers to Physical Exercise and Disability (B-PED) survey. Lack of interest, motivation, and energy as well as cost, lack of counseling on exercise by a physician, not having home equipment, and being too lazy were reported as barriers to exercise by all age groups. Those aged 35 to 54-years-old were more likely to report that cost, lack of transportation, having health concerns, not knowing where to exercise, and fear of leaving the home as barriers to exercise than those aged 18 to 34-years-old or 55-years-old and older. Overall, adults with TBI report multiple barriers to exercise, and these barriers vary by age.

## 1. Introduction

Based on data from the Centers for Disease Control and Prevention (CDC), over 2.5 million individuals suffer traumatic brain injury (TBI) annually in the United States [1]. TBI accounts for a high rate of morbidity and mortality. It is estimated that over 40% of the more than 3 million adults hospitalized with TBI in the United States will have long-term disability [2,3]. Older adults are the fastest growing population to suffer TBI and are the most likely age group to be admitted to the hospital following injury [4,5]. Older individuals with TBI have higher rates of disability and are less likely to be discharged to home from inpatient rehabilitation despite suffering less severe injuries than their younger counterparts [6,7].

Physical activity, which includes exercise training and daily physical activity participation, is an important adjuvant to medical treatment and is feasible for community-dwelling adults in recovery from moderate to severe TBI [8,9,10,11]. Participation in physical activity may positively affect cardiorespiratory fitness [9,12,13], fatigue [12,14], balance and mobility [15], mood [10,14,16,17,18], and cognition [17,18] following TBI. Current guidelines recommend that community-dwelling adults with a history of TBI perform 20 min of aerobic physical activity at low to moderate intensity at least 3 to 5 sessions per week along with strength, flexibility, and balance training [19]. Most individuals with TBI do not meet these recommendations [20,21], and their physical activity levels decline as early as one week following discharge from inpatient rehabilitation [21].

Both environmental factors and individual factors have been reported as barriers to physical activity for individuals following moderate to severe TBI [22,23]. Environmental barriers include lack of transportation and lack of accessible facilities to complete physical activity, whereas individual barriers include decreased endurance, self-consciousness in fitness centers, and lack of time [22]. Additionally, lack of knowledge regarding benefits of physical activity also contributes to decreased participation in exercise after moderate to severe TBI [24]. Hassett et al. [25] conducted a study of 30 community-dwelling individuals with recent severe TBI (mean time since injury of 2.3 months) who were enrolled in a home-based exercise program. Two-thirds of individuals were non-adherent to the exercise program, and older age, greater TBI severity, and lack of pre-injury aerobic exercise were significantly associated with decreased adherence to the exercise program, explaining 49% of the variance in their model [25].

Research in both TBI [26] and spinal cord injury (SCI) populations [27] found that persons injured later in life report less environmental barriers; however, study authors attributed this to avoidance of situations with barriers to physical activity participation rather than an actual reduction of barriers [27]. Franco et al. [28] conducted a systematic review of perspectives on physical activity participation in older adults. Lack of appropriate fitness instructors, costs, medical co-morbidities, lack of time, and apathy were frequently cited barriers to physical activity. Environmental barriers, such as lack of transportation, poor weather, living in an unsafe neighborhood, and lack of available exercise programs or equipment, were also reported [28].

Scientific evaluation of the studies on TBI-related environmental and individual barriers to physical activity and exercise participation is complicated by methodological differences in research design and execution with small sample sizes [22,24,25], heterogeneity in patient selection within studies with inclusion of adults with multiple sclerosis, SCI, stroke, and postpolio [29,30,31,32], and use of non-standardized outcome measures such as semi-structured interviews [24]. Additionally, there are no studies that investigate the difference in barriers to exercise with aging in individuals following TBI. The primary purpose of this study was to explore self-reported barriers to exercise faced by adults with TBI and to determine whether these barriers vary with age.

## 2. Materials and Methods

Following institutional review board approval we administered a mailed validated 28-item questionnaire on physical activity participation and structured exercise—the Barriers to Physical Exercise and Disability Survey (B-PED) developed by the National Center of Physical Activity and Disability [33] (Supplementary Materials). The developers of the B-PED validated the instrument among 53 African-American women in age from 18 to 64 years living with a severe physical disability according to the Americans with Disabilities Act and reported test-retest reliability coefficient of 0.76 for Cohen’s kappa and 0.86 for interrater reliability [33]. Additionally, the B-PED was validated for use in people with SCI [34]. Thirteen items had three choices: of which twelve had: “yes”, “no”, and “don’t know” and one item had “important”, “neither important nor unimportant” and “unimportant”. Fourteen items had two choices: of which thirteen had “true” and “false”, and one had “more” or “less” response. One item on type of exercise respondent would prefer to do had eight choices. Additional questions regarded demographic characteristics included race, gender, marital status, household income range, education level, assistive devices, employment status, length of time since TBI, exercise location preference, and age. 

Questionnaires were mailed in self-addressed stamped return envelopes to all 1,310 adults with moderate-severe TBI who have previously consented into the Carolinas Rehabilitation TBI Model Systems Database. Inclusion criteria were: (a) history of acquired brain injury due to trauma (traumatic brain injury was defined as “an alteration in brain function, or other evidence of brain pathology, caused by an external force” [35], (b) 18 years of age or older at the time of study participation, (c) TBI occurred at minimum 6 months prior to survey, (d) living in a private residence (non-nursing home or rest home), (e) able to read and complete the written survey, (f) able to give informed consent. Exclusion criteria were: (a) participant did not consent to the TBI Registry, (b) participant no longer part of the TBI Registry due to request to withdraw from the registry or death, (c) participant not able to read and complete the survey or need a care giver to complete. The study was carried out in accordance with the Declaration of Helsinki, and IRB approval was obtained at the study institution.

The letter inviting participation disclosed the study purpose and methodology. The written directions given to the participant were to “reflect on exercise that you have done since your traumatic brain injury.” The specific instructions were that “physical therapy is not considered exercise for this survey” [33]. Exercise program participation was defined as “some type of structured activity that is done on a regular basis such as walking, lifting weights, doing aerobics or riding a stationary bike” [33]. No prompts about study completion were sent to the participant database after the questionnaires were mailed out.

Participant ages were grouped into 18 to 34-years-old, 35 to 54-years-old, and 55-years-old and older. Statistical analyses were calculated using SAS Studio (version 3.7, SAS Institute, Cary, NC, USA). Data from the B-PED were analyzed using descriptive and summary statistics. Comparison of number of barriers reported per age group was determined using the analysis of variance procedure. Fisher exact and chi-square analyses were used to compare rate of individual barriers by age group. The level of significance was set at *p* < 0.05.

## 3. Results

### 3.1. Demographics

Within 2 weeks, 172 surveys were returned, and no additional surveys were returned after 2 weeks. Respondent demographic variables are shown in Table 1.

Significant differences exist in income and current work history based on age. There is also a significant difference in cause of TBI based on age. The majority of individuals (67.44%) suffered TBI as a result of motor vehicle accident. Falls were the next most common cause of injury (13.95%), and only those aged 35 to 54-years-old suffered TBI as result of fall (Table 1). There was no difference in self-report to liking exercise, desire to begin exercise program, having ever exercised, having exercised since TBI, and feel that exercise will help based on age group (Table 2).

### 3.2. Perceived Barriers to Exercise Participation by Age Group

Average total number of perceived barriers to exercise participation was 4.34 (standard deviation (SD) 3.34) for those 18 to 34-years-old, 4.78 (SD 2.46) for those 35 to 54-years-old, and 4.83 (SD 3.62) for those 55-years-old and older. There was no difference in average number of barriers to exercise participation reported per age group (*p*-value 0.64).

**Figure 1:**
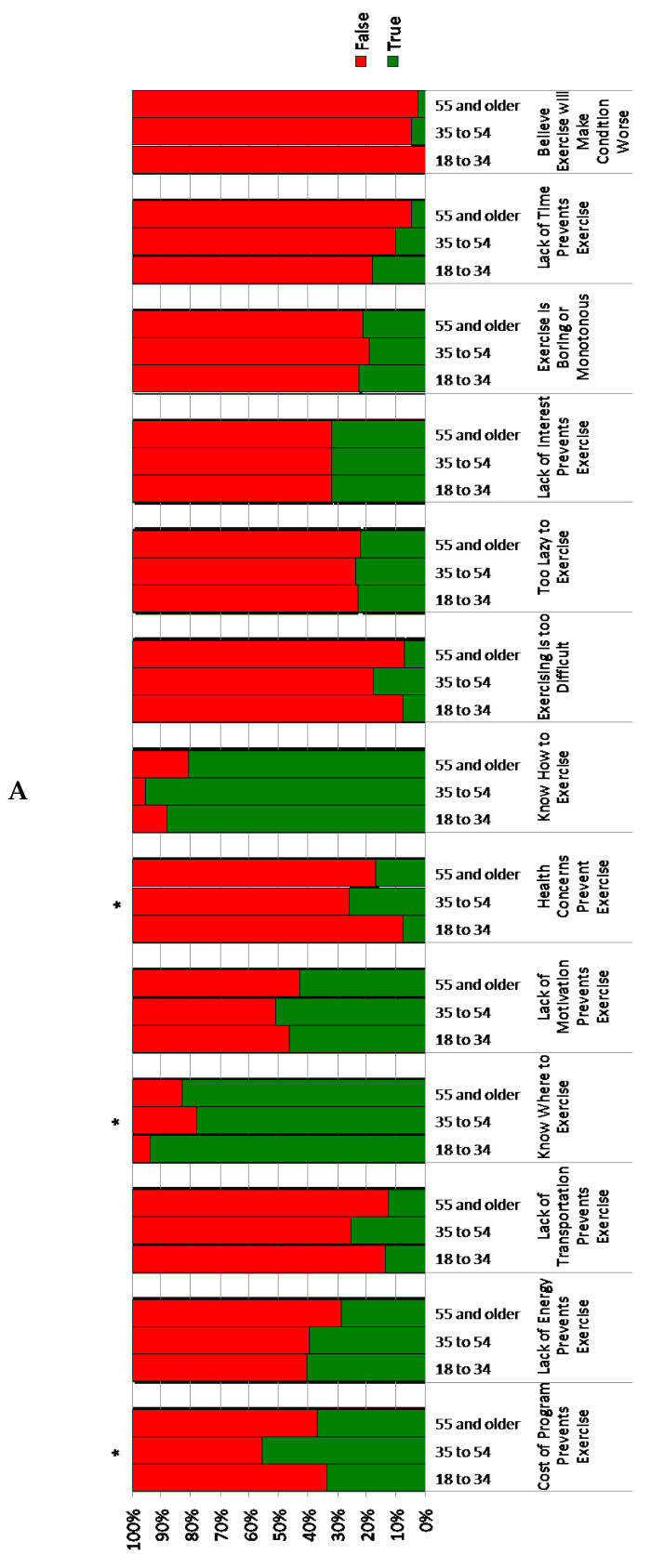

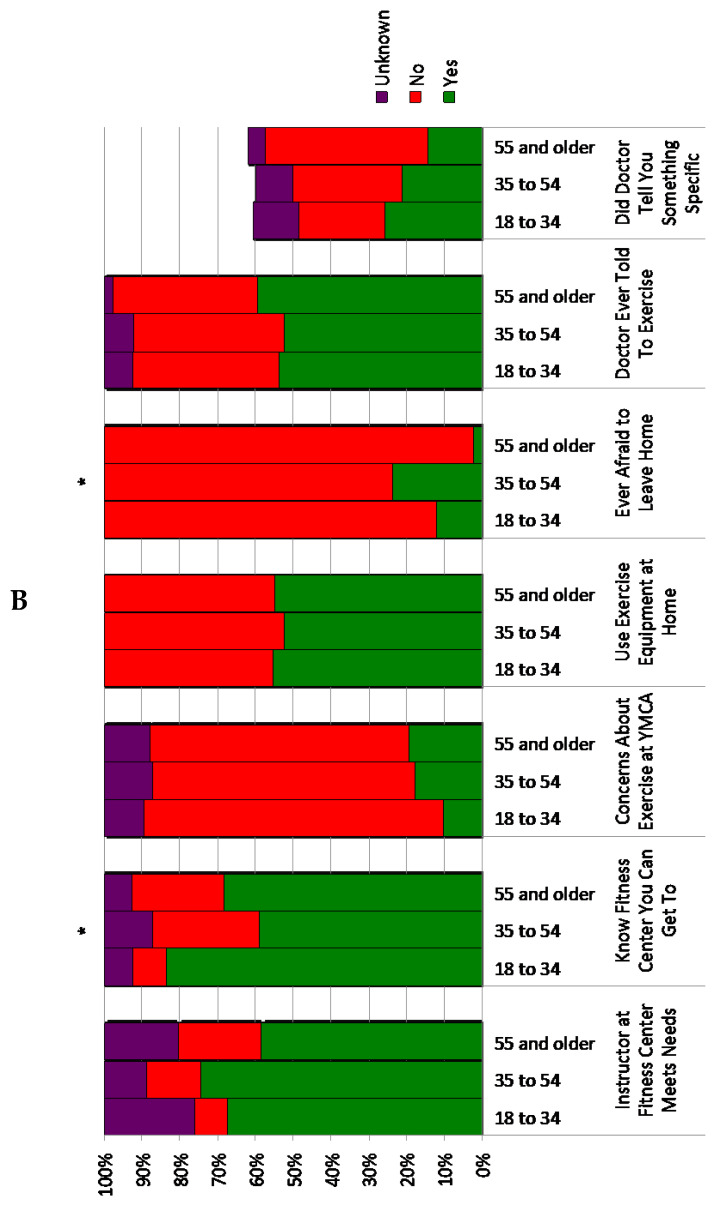
Reported barriers to exercise based on age. * *p*-value < 0.05. (**A**,**B**) represent different parts of the survey.

Perceived barriers to exercise participation listed by at least 20% of the age group are provided in descending order in Table 3. Overall, cost of program, lack of motivation, lack of energy, lack of interest, not having exercise equipment to use at home, not having a physician provide instruction to exercise, and feeling too lazy to exercise were perceived barriers to exercise participation for each age group (Table 3). Of these barriers, there was a significant difference in rate of cost of program being a barrier to exercise based on age (χ^2^ = 7.25, *p*-value = 0.02; Figure 1A). Cost of program was reported as a barrier for 55.56% of those aged 35 to 54-years-old but only in 33.33% and 36.59% of those aged 18 to 34-years-old and 55-years-old and older, respectively (Figure 1A, Table 3).

Over 20% of those aged 35 to 54-years-old reported 13 barriers to exercise participation compared with 9 in those aged 18 to 34-years-old and 11 in those aged 55-years-old and older (Table 3).

Ever afraid to leave the home, lack of transportation, not knowing where to exercise, and having health concerns that prevent exercise participation were only reported by at least 20% in the 35 to 54-year-old age group (Table 3). Of these barriers, there is a significant difference in report of being afraid to leave home (χ^2^ = 10.00, *p*-value = 0.00), health concerns preventing exercise (χ^2^ = 7.91, *p*-value = 0.01), and not knowing where to exercise (χ^2^ = 6.95, *p*-value = 0.03) based on age (Figure 1A,B).

There is a significant difference in rate of knowing how to access a fitness center based on age (χ^2^ = 10.9, *p*-value = 0.02). Over 80% of those aged 18 to 34-years-old know how to access a fitness center compared with 58.73% of those 35 to 54-years-old and 68.29% of those 55-years-old and older (Figure 1B). There is a trend towards higher belief that the trainer at the fitness center will not meet the needs with increased age, but this was not statistically significant (χ^2^ = 6.99, *p*-value = 0.13).

## 4. Discussion

The purpose of this study was to explore perceived barriers to participation in exercise post-TBI and determine if these perceived barriers differ based on age. There was no difference in number of barriers to exercise participation reported by individuals based on age group. Cost, lack of home equipment, and lack of counseling on exercise by physician were reported as barriers to exercise participation following TBI amongst all age groups. Additionally, each age group reported lack of motivation, energy, and interest as well as being “too lazy” as barriers to participation in exercise. We found a number of personal and environmental perceived barriers to exercise after TBI vary based on age, with those aged 35 to 54-years-old being more likely to report the following barriers than the other age groups: (1) the cost of the exercise program, (2) lack of transportation, (3) having health concerns that prevent exercise, (4) not knowing where to exercise, and (5) fear of leaving the home.

A theoretical framework underlying barriers to physical activity was conceptualized using the International Classification of Functioning, Disability and Health framework (ICFDH) [36,37]. The ICFDH framework is useful in conceptualizing barriers to exercise because it categorizes reported barriers to exercise into six domains: health conditions, body functions and structures, environmental factors, personal factors and participation [36]. The health conditions domain describes the condition under investigation. The body functions and structures domain addresses symptoms related to the condition, length of time living with the condition, or presence of co-morbidities. The environmental factors domain addresses physical and societal factors impacting physical activity participation. The personal factors domain addresses personal attributes and beliefs. A theoretical framework was developed for barriers to exercise in those following TBI based on age group (Table 4).

### 4.1. Personal Factors

Persons with TBI frequently experience fatigue, sleep-cycle dysregulation, anxiety, depression, apathy, aggression/irritability, and cognitive disorders [38,39,40]. Lack of motivation, decreased energy, lack of interest, and being “too lazy” to exercise were commonly reported barriers to exercise in all age groups following TBI (Table 3). Fatigue and decreased motivation have also been identified as barriers to physical activity and exercise in those with all types of disability [31,41,42]. Lack of time was only reported as a barrier by a small number of individuals with a trend towards decreased reporting of lack of time as a barrier with increased age (Figure 1A).

There is a significant difference in report of fear of leaving the home being a barrier to exercise based on age. Over 20% of those in the 35–54-year age group expressed fear over leaving the home, which was significantly higher than the other age groups. Prior research in those with SCI found only 6.9% reported fear of leaving home as a barrier to exercise [34]; however, 39% of African-American women with severe physical disabilities reported that fear of leaving the home was a barrier [33]. There is a high rate of anxiety after brain injury [38], and anxiety has previously been identified as a barrier to physical activity and exercise participation [9,21]. It is plausible that the fear to leave the home is related to higher perceived environmental barriers. In particular, those aged 35 to 54-years-old were more likely to report lack of transportation as barrier to exercise than the other age groups (Table 3); although, this was not a significant difference. Fear of leaving the home may also be related to perceived stigma faced by those with disabilities [29]. One study of young adults with childhood-onset physical disabilities found that some individuals with disability may feel uncomfortable or ashamed while doing physical activity [41].

Those aged 35 to 54-years-old are significantly more likely to report health concerns as a barrier to exercise (25.81%) than those aged 55-years-old and older (16.67%) or 18 to 34-years-old (7.46%) (Figure 1). Older age is associated with a higher rate of co-morbid conditions [43,44,45] and increased mortality [46,47]; therefore, it would be expected that health concerns would be a more frequently reported barrier with increasing age. Age-related differences in perceptions of health may explain why the middle age group was more likely to report health concerns as a barrier to exercise. A survey of 660 community-dwelling adults found that individuals 60-years-old and older were more likely to rate their health positively than younger age groups [48]. A longitudinal study of more than 2000 individuals aged 65-years-old and older also found a higher rate of positive self-report of health with increasing age [49].

### 4.2. Environmental Factors

In the present analysis, environmental factors were frequently cited as barriers to exercise after TBI in all age groups. Cost was a frequently reported barrier to exercise for our population, reported by over half of those in the middle age group and at least one-third of the young and older age groups. This finding is similar to prior research in those with physical disabilities that determined cost was a barrier to physical activity [31,41]; however, the rate of reporting cost as a barrier to exercise is higher in our population. In a study by Ellis et al., only 12.6% of those with physical disabilities reported that cost was a barrier to physical activity [31]. This finding may be due to the high rate of cognitive, psychiatric, and behavioral changes associated with TBI. Those with TBI face numerous difficulties maintaining employment, which is associated with decreased monthly income. Individuals with TBI face a high rate of unemployment [50,51], associated with a significant decrease in earned monthly income and increased reliance on public assistance [51]. In our sample, the majority of individuals had annual household income less than $50,000 per year, with a high rate of individuals reporting annual income of less than $20,000 per year (Table 1). In the present sample, those aged 35 to 54-years-old were significantly more likely to report cost as a barrier to exercise than the other age groups. They were also least likely to be employed and were most likely to have an annual income less than $20,000, which may explain this finding. The middle age group is also the group most likely to have family to support and other financial obligations that make cost a higher barrier to exercise. A small study in Malaysia investigating differences in barriers to exercise found that cost was cited as a barrier in significantly more middle-aged individuals than elderly individuals [52].

Roughly one-quarter of individuals in the 35 to 54-year-old age group reported lack of transportation as a barrier to exercise participation. Multiple prior studies also found access to transportation to be an issue for individuals recovering from TBI. One study found that lack of transportation was a major barrier to participation in an exercise program for those with TBI [9]. Corrigan and colleagues studied individuals aged 16–80+ with moderate to severe TBI and showed that the inability to drive oneself increased proportionally with increasing age [6]. Previous research by Devitt and colleagues revealed that less access to and availability of transportation predicted worse self-care and productivity outcomes among individuals with moderate to severe brain injury [53]. Lack of transportation has also been linked to negative employment outcomes. A 2013 study by Forslund et al. found that driving a vehicle was a significant predictor of employment 2 years after TBI in individuals 16–55 years of age [54]. Lack of transportation has been noted as a barrier to physical activity in those with physical disabilities [31,41]. Taken together, the studies mentioned above underline the importance that access to transportation has on the ability of individuals who have suffered a TBI to return to active community participation.

Not knowing where to exercise was a frequently cited barrier to exercise participation, reported by about a third (28.5%) of the respondents in the 35 to 54-year age group and nearly a quarter (23.3%) of the respondents in the 55 years and older age group. These results are similar to a prior study that found going to a fitness center was a barrier to participation in an exercise program after TBI [9]. Additionally, one study found one-third of respondents with SCI (mean 44.1 years of age) did not know where to exercise [34], and two other studies in those with physical disabilities found that lack of an available fitness center was a barrier to physical activity [31,41]. It is unclear whether awareness or utilization of community-based fitness resources translate to heightened physical activity participation levels for community-dwelling older adults [55]. There is some evidence that for older adults with peripheral vascular disease home based exercise programs may be superior to fitness center based exercise programs in terms of adherence to exercise [56]; however, this has not been investigated in adults following TBI. The present study also found not using exercise equipment at home was listed as a top-3 barrier reported by greater than 40% of individuals in all age groups (Table 3).

Not being told to exercise by a physician was reported as a barrier in over one-third of individuals in each age group. Further, for those who were instructed to exercise, lack of specific instructions was reported by at least one-fifth of individuals (Table 3). Overall, physician counseling for exercise is limited in the United States. Wee et al. (1999) [57] investigated the rate of physician counseling using the National Health Interview Survey (NHIS). Of the 9299 individuals who responded, only 34% reported being counseled on exercise at the last physician visit [57]. More recently, Ahmed et al. (2017) [58] also found a low rate of physician counseling on exercise on the NHIS, but there is a trend towards increased physician counseling on exercise. In their study, only 22.9% reported being counseled on exercise by their physician within the past 12 months in 2000, which increased to 33.6% in 2010 [58]. Those with higher annual income, health insurance, and higher education levels were more likely to receive counseling on exercise [57,58]. Additionally, those who were overweight/obese and had cardiac disease or diabetes were most likely to report counseling on exercise [57], highlighting a missed opportunity to discuss exercise to prevent these medical co-morbidities. A systematic review found multiple barriers to physician counseling on physical activity, namely lack of time, reimbursement, and training on physical activity counseling [59]. Focused education on physical activity counseling during medical school improves comfort with counseling about physical activity [60], but further research is needed to determine whether this will lead to changes in clinical practice.

### 4.3. Strengths/Limitations

This is the largest study investigating barriers to exercise participation in individuals living with TBI, and to the authors’ knowledge, is the first to investigate differences based on age. Still, a limitation of the current investigation is the small sample size. In order to achieve a 95% confidence level with a 5% margin of error, a sample size of 298 participants would have been required. The survey response rate was only 13.1%; however, this is within the expected response rate for mail-in surveys in individuals following TBI [61,62,63]. A strength of this study is the use of a structured survey instrumentation to assess barriers to exercise participation in persons with disability [33]. This allowed for survey of a larger number of individuals than other studies investigating barriers in persons with TBI [22,24,25]. The survey chosen was one that had been previously utilized in persons with disability [33]. The primary limitation is that this is a single center study where all individuals are part of a local TBI registry comprised of individuals who have been discharged after inpatient rehabilitation. The sample was comprised of those who had voluntarily returned the survey. This may lead to a selection bias where the study sample may be more likely to participate in physical activity; thus, the results may not be generalizable to the population as a whole. Additionally, by use of a self-reported survey we are only able to determine perceived barriers to exercise reported by individuals with TBI; we were unable to determine whether these barriers were physically present. Use of the survey instrument also limits us to studying the barriers that were reported by individuals with TBI; therefore, we may miss some additionally barriers that were not part of the survey. We did not assess for participant self-efficacy for exercise, which has been highly associated with physical activity in those with disabilities [32,41,42]. We did not assess respondents’ physical activity levels. This needs to occur in future studies in order to evaluate whether the number of perceived barriers to physical activity participation are associated with age and physically active/physically sedentary status. We used a cross-sectional study design. Cross sectional survey designs are quick, easy and cheap to design, however, only allow an association of barriers to physical activity participation with age, and not causation, to be inferred. Additionally, the findings require self-reporting of barriers, which may lead to recall bias or potential under or over-reporting of barriers to exercise participation. We feel that a prospective observational study where investigators following individuals with TBI into the community to monitor exercise and associated barriers would be helpful as we would eliminate the multiple limitations of self-report described above. The literature is divided between studies that use the terms “physical activity” and “exercise,” which limits our ability to compare our results directly with others. This investigation uses the term exercise as it is the terminology used in the B-PED, and it is possible that studies using the term “physical activity”” may produce somewhat different results based on participants perceptions of what these activities mean to them, despite investigators best efforts to define the terms to the target population a priori. Finally, we only listed barriers reported by at least 20% of each age group in Table 3 to highlight those barriers that were experienced by a high enough proportion of the population to be clinically relevant.

## 5. Conclusions

Individuals with TBI report a number of barriers to exercise participation that vary based on age group. All groups reported that costs, lack of home equipment, lack of physician counseling on exercise, decreased motivation, lack of interest, and poor energy as being barriers. There was no difference in number of barriers to exercise based on age group; however, those aged 35 to 54-years-old were more likely to cite costs, health concerns, lack of transportation, fear of leaving the home, and not knowing where to exercise as barriers than those aged 18 to 34-years-old or 55-years-old and older. The present data are preliminary, but if verified in a larger data set, will identify barriers to be addressed in any program designed to encourage physical activity participation in the aging TBI population. Future studies should additionally investigate the impact of additional resources, such as addition of a case manager skilled in individuals with TBI, in order to overcome barriers to exercise participation.

## Figures and Tables

**Table 1:** Demographics Values are total (percentage).

Participant Characteristic	18 to 34 Years (*n* = 67)	35 to 54 Years (*n* = 63)	55 Years and Older (*n* = 42)	*p*-Value
Female sex	25 (37.31%)	23 (36.51%)	15 (35.71%)	0.9856
Cause of TBI				<0.0001 *
-- Motor Vehicle	66 (98.51%)	24 (38.10%)	26 (61.90%)	
-- Fall	0 (0%)	24 (38.10%)	0 (0%)	
-- Pedestrian	0 (0%)	2 (3.17%)	13 (30.95%)	
-- Sport	0 (0%)	6 (9.52%)	0 (0%)	
-- Assault/GSW	0 (0%)	6 (9.52%)	0 (0%)	
-- Other/Unknown	1 (1.49%)	1 (1.59%)	3 (7.14%)	
>5 years since TBI	37 (56.06%)	31 (49.21%)	28 (66.67%)	0.2101
Household income				0.0009 *
-- <$20,000/year	28 (45.90%)	34 (55.74%)	9 (23.68%)	
-- $20,000–$49,999/year	15 (24.59%)	16 (26.23%)	12 (31.58%)	
-- $50,000–$99,999/year	17 (27.87%)	5 (8.20%)	15 (39.47%)	
-- >$100,000/year	1 (1.64%)	6 (9.84%)	2 (5.26%)	
Employed				<0.0001 *
-- Full/part time	25 (37.88%)	9 (14.29%)	9 (21.95%)	
-- Volunteer	2 (3.03%)	1 (1.59%)	6 (14.63%)	
-- Student	9 (13.64%)	3 (4.76%)	0 (0%)	
-- Not employed	30 (45.45%)	50 (79.37%)	26 (63.41%)	

TBI = traumatic brain injury; GSW = Gunshot wound; * Significant based on *p*-Value < 0.05.

**Table 2:** Desire to exercise based on age group.

Desire to Exercise	18 to 34 Years (*n* = 67)	35 to 54 Years (*n* = 63)	55 Years and Older (*n* = 42)	*p*-Value
Like to Exercise	58 (86.57%)	44 (73.33%)	29 (69.05%)	0.1729
Like to Begin Exercise Program	42 (63.64%)	39 (61.90%)	21 (53.85%)	0.3831
Ever Exercised	66 (98.51%)	57 (90.48%)	41 (97.62%)	0.1917
Exercise since TBI	62 (92.54%)	50 (79.37%)	34 (82.93%)	0.2522
Feel exercise will help	53 (79.10%)	45 (70.97%)	36 (85.37%)	0.2004

TBI = traumatic brain injury.

**Table 3:** Perceived Barriers to Exercise Reported by at Least 20% of Individuals Listed in Order Reported.

18 to 34 Years (*n* = 67)	35 to 54 Years (*n* = 63)	55 Years and Older (*n* = 42)
Lack of Motivation Prevents Exercise (46.7%)	Cost of Program Prevents Exercise (55.56%) *	Do Not Use Exercise Equipment at Home (45.00%)
Do Not Use Exercise Equipment at Home (44.78%)	Lack of Motivation Prevents Exercise (50.79%)	Lack of Motivation Prevents Exercise (42.86%)
Lack of Energy Prevents Exercise (40.30%)	Do Not Use Exercise Equipment at Home (47.62%)	Doctor Never Told to Exercise (38.1%)/Something Specific (42.86%)
Doctor Never Told to Exercise (38.81%)/Something Specific (22.73%)	Doctor Never Told to Exercise (39.68%)/Something Specific (29.03%)	Cost of Program Prevents Exercise (36.59%) *
Cost of Program Prevents Exercise (33.33%) *	Lack of Energy Prevents Exercise (39.68%)	Lack of Interest Prevents Exercise (31.71%)
Lack of Interest Prevents Exercise (31.82%)	Lack of Interest Prevents Exercise (31.75%)	Lack of Energy Prevents Exercise (28.57%)
Too Lazy to Exercise (22.73%)	Do Not Know Fitness Center That Can Get to (28.57%) *	Did Not Know Fitness Center That Can Get To (24.39%) *
Exercise is Boring or Monotonous (22.39%)	Health Concerns Prevent Exercise (25.81%) *	Too Lazy to Exercise (21.95%)
	*Lack of Transportation Prevents Exercise (25.4%)*	Fitness Instructor Does Not Meet Needs (21.95%)
	Ever Afraid to Leave Home (23.81%) *	Exercise is Boring or Monotonous (21.43%)
	Too Lazy to Exercise (23.81%)	
	Do Not Know Where to Exercise (22.22%) *	

* Significant difference between groups (*p*-value < 0.05). Italics indicate barrier only listed by at least 20% of sample in those aged 35 to 54-years-old.

**Table 4:** Perceived barriers by age among 172 adults with moderate to severe traumatic brain injury according to International Classification of Functioning, Disability and Health framework (ICFDH). International Classification of Functioning, Disability and Health (ICFDH) theoretical framework for barriers to exercise in adults with TBI. ✓ = perceived barrier reported by at least 20% of the age group. Red rectangle indicates the top barrier for the age group. Yellow rectangle is the second most reported barrier for the age group, and green rectangle is the third most frequent barrier reported by the age group.

	Age Group		
ICFDH Factor, Barrier	18–34	35–54	55+
1. Physical Environmental Factors			
Cost of the program	✓	✓	✓
Do not use home equipment	✓	✓	✓
Do not know fitness center to get to	✓	✓	✓
Do not know where to exercise		✓	
Fitness instructor does not meet needs			✓
Lack of transportation		✓	
2. Social Environmental Factors			
Lack of encouragement from doctor	✓	✓	✓
3. Body Function/Structures Factors			
Lack of motivation	✓	✓	✓
Lack of energy	✓	✓	✓
Too lazy to exercise	✓	✓	✓
Health concerns prevent exercise		✓	
Too afraid to leave the home		✓	
4. Personal Factors (Beliefs)			
Lack of interest	✓	✓	✓
Exercise is boring		✓	

## Supplementary Materials

The following are available online at http://www.mdpi.com/2411-5142/3/3/47/s1.
