# Supplementary material for: Perceived Barriers to Exercise in Adults with Traumatic Brain Injury Vary by Age
Source: J Funct Morphol Kinesiol. 2018 Sep 14;3(3):47. doi: 10.3390/jfmk3030047 (PMC7739410; doi:10.3390/jfmk3030047)
Supplement: Supplementary file 1 [file jfmk-03-00047-s001.pdf]

Traumatic Brain Injury Exercise Habits Survey

Thank you for participating in this survey. The purpose of the survey is to identify potential barriers to physical activity individuals with traumatic brain injury (TBI) experience. Physical activity is essential to overall health and can be helpful in controlling weight, reducing risk of heart disease and some cancers, strengthening bones and muscles, and improving mental health. We would like to determine what prevents individuals with TBI from being physically active and thus unable to benefit from this involvement. Thank you for answering the following questions.

By completing this survey, you are giving consent for us to use this data. All data is confidential. No identifying data, such as name, date of birth, or address, are collected for this study.

❑ Yes, consent is given

1. Who is completing this survey? ❑ Individual with TBI ❑ Caregiver of person with TBI*

** If you are completing the survey as the caregiver, please answer the questions as they refer to the **individual with TBI**.*

2. When did your TBI occur?

❑ less than 1 year ago ❑ 2-3 years ago ❑ 4-5 years ago

❑ 1-2 years ago ❑ 3-4 years ago ❑ more than 5 years ago

3. What was the specific cause of your TBI (example: fall, car accident, struck by a vehicle)?

____

____

____

4. What State and County do you live in? ❑ NC ❑ SC

County ______________________________

5. What is your age?

❑ 18 – 24 years ❑ 36 – 45 years ❑ 56 – 65 years ❑ 75 years or older

❑ 25 – 35 years ❑ 46 – 55 years ❑ 66 – 75 years

6. What is your gender? ❑ Female ❑ Male

7. What is the closest estimate for your annual household income?

- ❑ less than $20,000
- ❑ $50,000 - $99,999
- ❑ $150,000 - $199,999
- ❑ $20,000 - $49,999
- ❑ $100,000 - $149,999
- ❑ $200,000 or more

8. What level of education did you complete?

- ❑ less than high school diploma
- ❑ college degree
- ❑ high school diploma
- ❑ graduate degree

9. What assistive devices do you use to help you move around? (Check all that apply)

- ❑ None
- ❑ Walker
- ❑ Wheelchair
- ❑ Cane
- ❑ Leg or ankle brace
- ❑ Other___________________________

10. Where would you prefer to exercise?

- ❑ at home
- ❑ at a rehabilitation center
- ❑ does not matter
- ❑ at a fitness center
- ❑ in my neighborhood

11. Please check the answer that best describes your work situation.

- ❑ I work full time
- ❑ I am a volunteer
- ❑ I am a student
- ❑ I work part time
- ❑ I am not working at this time

12. Do you feel irritable or frustrated since your injury? ❑ No ❑ Yes

Directions: An exercise program is participation in some type of structured activity that is done on a regular basis such as walking, lifting weights, doing aerobics or riding a stationary bike. In the following section we would like you to reflect on exercise that you have done since your traumatic brain injury. **Physical Therapy is not considered exercise for this survey.**

13. Do you like to exercise? ❑ No ❑ Yes ❑ Don't know

14. What type of exercise would you prefer to do?

- ❑ Water exercise
- ❑ Walking
- ❑ Exercise to strengthen muscles
- ❑ Tai Chi
- ❑ Exercise to improve balance
- ❑ Team sports
- ❑ Meditation; yoga
- ❑ Aerobic exercise, i.e. dancing, running, biking

15. Would you like to begin an exercise program? ❑ No ❑ Yes ❑ Don't know

16. Have you ever exercised? ❑ No ❑ Yes ❑ Don't know

17. Have you exercised since your traumatic brain injury? ❑ No ❑ Yes ❑ Don't know

18. Do you feel like an exercise program could help you? ❑ No ❑ Yes ❑ Don't

19. Do you feel that an exercise instructor in a fitness center would know how to set up an exercise program to meet your needs? ❑ No ❑ Yes ❑ Don't know

20. Do you know of a fitness center that you could get to? ❑ No ❑ Yes ❑ Don't

21. Would you have any concerns about exercising in a facility like a YMCA?

❑ No ❑ Yes ❑ Don't know

22. Do you have any exercise equipment at home that you use? ❑ No ❑ Yes ❑ Don't

23. Are you ever afraid to leave your home? ❑ No ❑ Yes ❑ Don't know

24. Has your doctor ever told you to exercise? ❑ No ❑ Yes ❑ Don't know

25. If yes, did your doctor ever tell you to do anything specific? ❑ No ❑ Yes ❑ Don't know

26. Did you exercise more or less after your traumatic brain injury? ❑ More ❑ Less

27. On a scale of 1 to 3 with 1 being important and 3 being unimportant, how important is exercise to you? ❑ 1 (important) ❑ 2 (neither important nor unimportant) ❑ 3 (unimportant)

28. The cost of an exercise program prevents me from exercising. ❑ True ❑ False

29. Lack of energy prevents me from exercising. ❑ True ❑ False

30. Lack of transportation keeps me from exercising. ❑ True ❑ False

31. I know where to exercise. ❑ True ❑ False

32. Lack of motivation prevents me from exercising.	☐ True	☐ False
33. Health concerns prevent me from exercising.	☐ True	☐ False
34. I know how to exercise.	☐ True	☐ False
35. Exercising is too difficult.	☐ True	☐ False
36. I am too lazy to exercise.	☐ True	☐ False
37. Lack of interest prevents me from exercising.	☐ True	☐ False
38. Exercise is boring or monotonous.	☐ True	☐ False
39. Lack of time prevents me from exercising.	☐ True	☐ False
40. I believe exercise will make my condition worse.	☐ True	☐ False

Thank you for completing the survey. We value your participation!
Please return the survey to the following address in the self addressed and self stamped envelope enclosed.
